# Low‐operation voltage conductive‐bridge random access memory based on amorphous NbS_2_

**DOI:** 10.1002/smo.20230008

**Published:** 2023-09-22

**Authors:** Bojing Lu, Dunan Hu, Min Wu, Ruqi Yang, Yuying Ding, Jingyun Huang, Qinghua Zhang, Zhizhen Ye, Yang Hou, Jianguo Lu

**Affiliations:** ^1^ State Key Laboratory of Silicon and Advanced Semiconductor Materials School of Materials Science and Engineering Zhejiang University Hangzhou China; ^2^ Zhejiang Artiking Group Co., LTD Hangzhou China; ^3^ Wenzhou Key Laboratory of Novel Optoelectronic and Nano Materials Institute of Wenzhou Zhejiang University Wenzhou China; ^4^ Key Laboratory of Biomass Chemical Engineering of Ministry of Education College of Chemical and Biological Engineering Zhejiang University Hangzhou China

**Keywords:** conductive‐bridge random access memory, low voltage operation, NbS_2_, resistive switching

## Abstract

Amorphous NbS_2_ was proposed as the resistive switching (RS) layer for conductive‐bridge random access memory (CBRAM) for the first time, with Cu and Au as the top and bottom electrodes, respectively. NbS_2_ films were prepared at room temperature, which exhibited an amorphous structure and did not crystalize even annealed at 500°C, showing good thermal stability. The amorphous NbS_2_ CBRAM devices present stable bipolar non‐volatile RS characteristics. Repetitive RS behavior is demonstrated in amorphous NbS_2_ CBRAMs. The operating voltage during all RS cycles is less than 1 V, demonstrating that the NbS_2_ CBRAM is a low‐operation voltage memory device. The distribution of the high and low resistive state resistance is relatively concentrated, and the on‐off ratio has been kept above 100, offering a sufficient data read/write window. The formation and fracture of the Cu metal conductive filament is considered to be the RS mechanism by analyzing the dependence of current and voltage in logarithmic coordinates. Our study demonstrated that amorphous NbS_2_ is a promising material for low‐operation voltage CBRAM.

## Graphical abstract

Amorphous NbS_2_ films were proposed for producing CBRAMs, presenting stable bipolar resistive switching characteristics with operation voltage below 1 V and HRS/LRS ratio up to 100. Repetitive resistive switching behavior is demonstrated in *a*‐NbS_2_ CBRAM devices. The electrochemical redox reaction of Cu, as well as the formation and fracture of Cu metal CF, is the resistive switching mechanism.

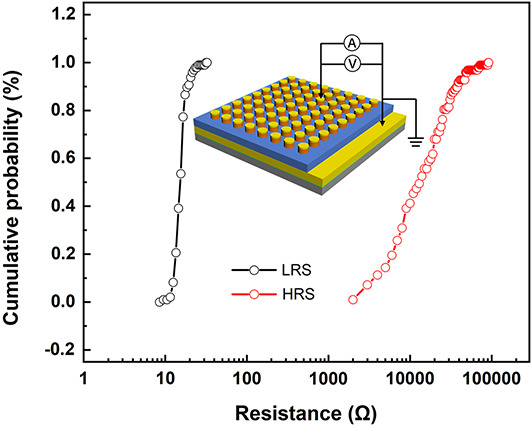

## 1 INTRODUCTION

The advent of high‐performance computing and the big data era has put forward higher and higher requirements for data storage and processing. Mainstream storage technologies are based on charge storage mechanisms: static random access memory (SRAM) stores charges in the storage nodes of cross‐coupled inverters, dynamic random access memory (DRAM) stores charges in cell capacitors, and FLASH stores charges in the floating gate dielectric layer of floating gate transistors. All of these charge‐based memory technologies face the challenge of scaling to 10 nm or more advanced process nodes, because the charges stored at the nanometer level are easily lost, resulting in a decline in memory performance, reliability, and noise tolerance. In this context, the industry is actively researching and developing new memory technologies based on non‐charge storage, hoping to completely change the current mainstream memory hierarchy. Resistive switching random access memory (ReRAM) has attracted intensive interest because of its great potential for novel non‐volatile memory applications and neuromorphic computing applications.[^1^, ^2^, ^3^, ^4^]

ReRAM is a device that uses changes in the resistance to store data, which are called resistive switching (RS) behavior.[^5^, ^6^, ^7^, ^8^, ^9^] Based on the metal conductive filament (CF) to realize the RS, the conductive‐bridge ReRAM (CBRAM) is a very promising one in the next generation of non‐volatile memory.[^10^, ^11^] The diameter of the resistance wire can be only a few nanometers or even sub‐nanometers. The low‐resistance state of this RRAM does not depend on the electrode area, so it has good potential for device scaling. Metal cations can migrate quickly in the RS dielectric layer, and the thickness of the dielectric layer only needs to be tens of nanometers to meet the storage requirements, so CBRAM devices have extremely fast RS speeds. The resistive switch layer material of the CBRAM device can be selected in a wide range, and the device performance will also change accordingly. The appropriate resistive switch layer dielectric material can be selected according to practical needs. CBRAM resistive memory is also very competitive in terms of cycle endurance and data retention characteristics, so conductive bridge resistive memory has always been the focus of academic research.

Although great progress has been made in the research and development of CBRAM, there are still many challenges to be solved. In previous reports, RRAM generally requires several volts of operating voltage to switch the device between different resistance states.^[12]^ Higher operating voltage will cause the device to consume a lot of power and generate heat, affecting the stability of the device. In particular, with the improvement of complementary metal oxide semiconductor (CMOS) process, the driving current that the transistor can provide also decreases.^[13]^ Although it is possible to increase the driving current by increasing the gate voltage of the transistor, a large gate voltage will inevitably introduce reliability problems for the gate dielectric. Therefore, the low‐voltage operating characteristics are a necessary condition for the continuous scaling of ReRAM, and how to reduce the operating voltage is still a key point in the research of advanced ReRAM. ReRAMs operated at low voltages have also been the focus of researchers.[^6^, ^14^, ^15^, ^16^]

The resistance switching performance of the CBRAM device depends on the material of the resistance switching layer. Materials that have been studied as RS layers include typical chalcogenides (such as GeSe,^[17]^ ZnS,^[18]^ MoSe,^[19]^ MoS,[^20^, ^21^] etc.) and various binary oxides (such as Ta_2_O_5_,[^22^, ^23^] ZnO,^[24]^ SiO_2_,[^25^, ^26^, ^27^] ZrO_2_,^[28]^ HfO_2_,[^29^, ^30^] etc.). Among them, the CBRAM using the chalcogenide as the resistive switch layer shows a lower operating voltage because the sulfide or selenide used as the resistive switch layer is usually an amorphous structure, which presents fast cation transport channels.^[3]^ Amorphous Nb_2_O_5_ and NbSe_2_ CBRAMs have been reported with ultra‐low operating voltages, where Nb_2_O_5_ exhibited multilevel RS characteristics and NbSe_2_ demonstrated the self‐repairable behavior.[^6^, ^7^] Oxygen, sulfur, and selenium are all VI A group elements, and transition metal sulfides are also typical materials used in CBRAM. What will happen if niobium sulfide is used as the switching layer in CBRAM? However, the NbS_2_‐based conductive bridge resistive memory has not been reported yet. In this work, NbS_2_ is used as the resistive switch layer for the first time, and a CBRAM device with Cu and Au as the top and bottom electrodes, respectively, is designed to explore its RS performance.

## 2 EXPERIMENT

**FIGURE 1:**
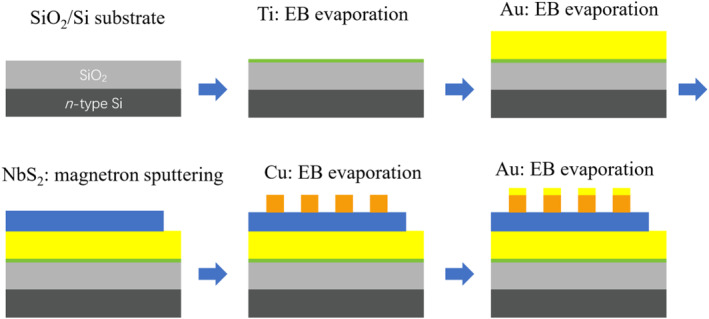
The preparation flow chart and device structure diagram of *a*‐NbS_2_ CBRAM. CBRAM, conductive‐bridge random access memory.

NbS_2_ thin films are prepared by the magnetron sputtering technique using NbS_2_ ceramic target with high purity of 99.99%. Quartz glass and SiO_2_/Si wafers were used as substrates. Samples grown on quartz substrates were used to perform some material characterizations. In addition to deposition NbS_2_ films for material characterization, the SiO_2_/Si silicon wafer substrate is used as a substrate for carrying CBRAM devices. Various substrates were ultrasonically cleaned with acetone, ethanol, and deionized water for 30 min sequentially, and then stored in absolute ethanol. The substrate was blown dry with high‐purity nitrogen before use, and then placed in the magnetron sputtering sample chamber. During the sputtering process, the temperature was room temperature, the atmosphere was pure argon, the pressure was 2 Pa, the sputtering power was 30 W, and the sputtering time was 60 min. The fabrication process of the NbS_2_ CBRAM device is shown in Figure 1. Firstly, a thin layer of metal Ti (∼10 nm) is deposited on the dry and clean SiO_2_/Si substrate by electron beam evaporation to enhance the adhesion between the metal Au (bottom electrode) and the substrate. Secondly, electrochemically inert metal Au was then grown as the bottom electrode using electron beam evaporation. Thirdly, NbS_2_ film (∼100 nm) was then deposited using magnetron sputtering. Electron beam evaporation was then used to grow electrochemically active metal Cu as the top electrode. The top electrode is a circle with a diameter of around 300 μm. A shadow mask is used during electron beam evaporation to define the size, position and number of top electrodes. Each top electrode corresponds to a discrete CBRAM device. Finally, a layer of Au was deposited to protect Cu from oxidation.

The surface and cross‐sectional images of the NbS_2_ films were carried out by a field emission scanning electron microscope (FESEM, Hitachi S4800). Elemental mapping was employed using an X‐ray energy spectrometer (EDS) attached to the SEM. The surface roughness was studied by atomic force microscopy (AFM, Brukerr Dimension Icon). The elemental distributions and chemical bonding states were investigated by X‐ray photoelectron spectroscopy (XPS, Thermo Scientific K‐Alpha). Also, the study of the crystal structure of NbS_2_ was conducted using x‐ray diffraction (XRD, Empyrean 200895); The prepared NbS_2_ CBRAM device uses a Keithley 4200A semiconductor parameter instrument to test the RS performance of the CBRAM device.

## 3 RESULTS AND DISCUSSION

**FIGURE 2:**
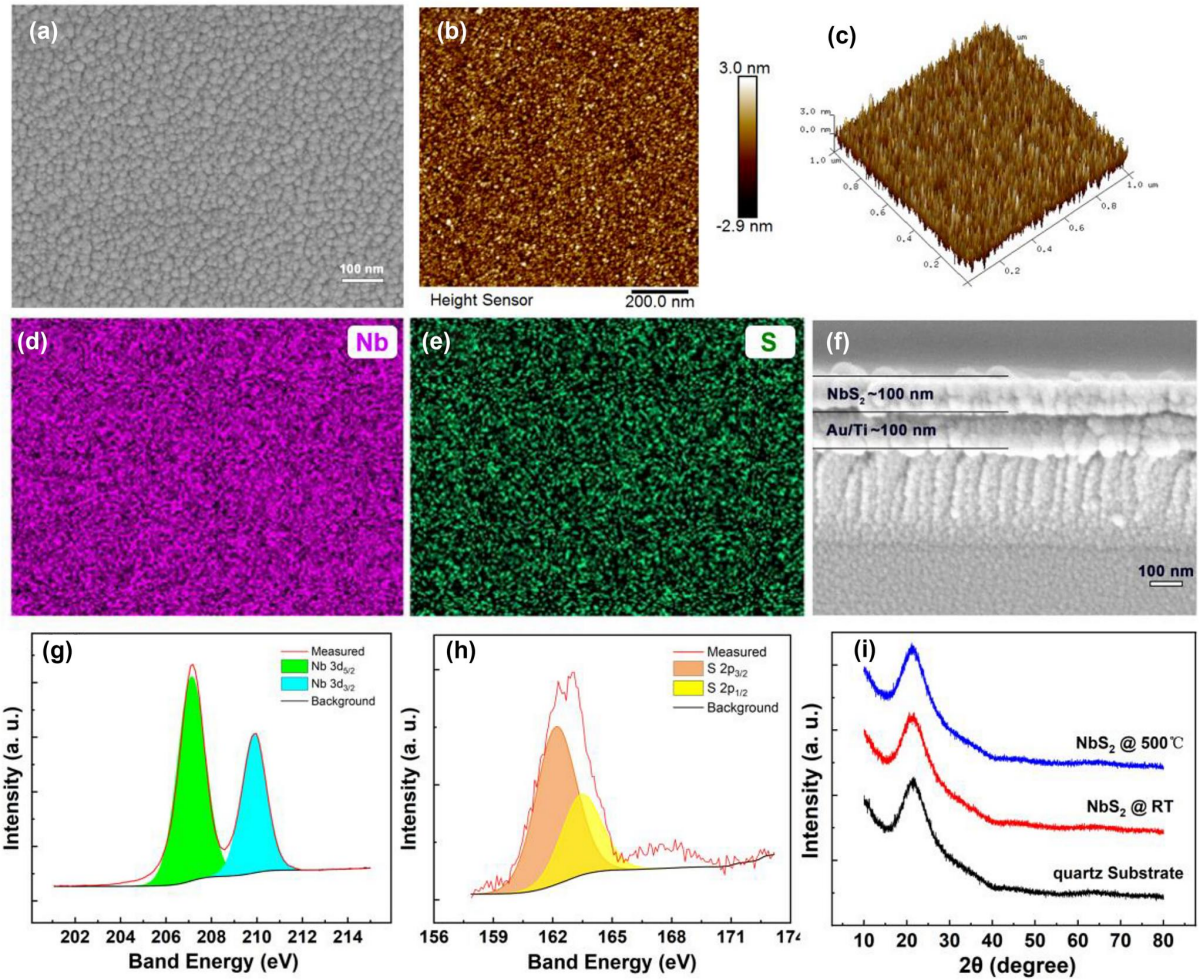
(a) SEM image of the surface of the NbS_2_ film; (b) the planar image and (c) the three‐dimensional image of the NbS_2_ surface obtained from the AFM test; the EDS elemental mapping images of the (d) niobium and (e) sulfur; (f) SEM image of the cross‐section of the NbS_2_ film; XPS spectra of the (g) Nb 3d and (h) S 2p; (j) the XRD patterns of the NbS_2_ film and the quartz substrate. AFM, atomic force microscopy; XPS, X‐ray photoelectron spectroscopy; XRD, x‐ray diffraction.

Figure 2a is the SEM image of the surface of the NbS_2_ film, it can be seen that the film has a dense, flat and smooth surface without any visible voids and defects. Figure 2b,c are the planar image and the three‐dimensional image of the NbS_2_ surface obtained from the AFM test, respectively. AFM can obtain the roughness and three‐dimensional topography of the film surface. The surface fluctuation of the NbS_2_ film prepared by magnetron sputtering does not exceed 6 nm. Calculated according to the test results, the root mean square roughness (Rq) of the film surface is only 0.858 nm, indicating that the films are smooth. A flat film surface is conducive to the preparation of the device, and it is also of great benefit to the consistency of the device performance. In order to characterize the surface element distribution of the NbS_2_ film, we used an X‐ray energy spectrometer attached to the SEM equipment to carry out the EDS elemental mapping test. Figure 2d,e are the EDS elemental mapping images of niobium and sulfur, respectively. As shown in the results, the sulfur and niobium have a uniform and continuous distribution on the film, and there is no element segregation and aggregation, suggesting that NbS_2_ film exhibits excellent uniformity. Figure 2f shows the cross‐section SEM image of the NbS_2_ film with uniform thickness around 100 nm. The XPS spectra of the Nb 3d and S 2p of NbS_2_ film are shown in Figure 2g,h respectively, which were calibrated with reference to the C 1s peak. The binding energy (BE) values for the most intense peaks of Nb 3d and S 2p were found to be 207.1 eV (Nb 3d_5/2_) and 162.1 eV (S 2p_3/2_), which correspond to Nb^4+^ and S^2−^.

As mentioned above, the crystallization of the resistive switch material has a great influence on the operating voltage of the CBRAM. The conductive bridge RRAM with an amorphous material as the resistive layer tends to have a lower operating voltage because its short‐range disorder can provide fast migration channels for cations.^[3]^ We used XRD to study the crystallization of NbS_2_ films prepared by magnetron sputtering. Figure 2i shows the XRD patterns of the NbS_2_ film and the quartz substrate. The black curve is the XRD pattern of the quartz substrate, and the red is the X‐ray diffraction pattern of the NbS_2_ film deposited on the quartz substrate at room temperature. The XRD spectrum of the NbS_2_ grown by magnetron sputtering is the same as that of the quartz substrate. There is only a halo peak near 21°, which is contributed to the quartz substrate, and no diffraction peak belonging to NbS_2_ appears, indicating that the NbS_2_ film grown by magnetron sputtering is amorphous.^[31]^ In order to further verify the thermal stability of the NbS_2_ thin film amorphous structure, we thermally annealed the NbS_2_ thin film and then carried out XRD test on the NbS_2_ film. The blue curve in Figure 2g is the XRD pattern obtained after NbS_2_ was annealed at 500°C for 30 min, and the film was still amorphous. It shows that NbS_2_ thin film has good thermal stability. This is of great help to the cycle stability of the device. Because the crystallization of the material has a great influence on its resistance. Heat is generated when the device is in operation, and the crystallization of the resistive variable layer will affect its resistance value, which has a great adverse effect on the resistive variable memory.

**FIGURE 3:**
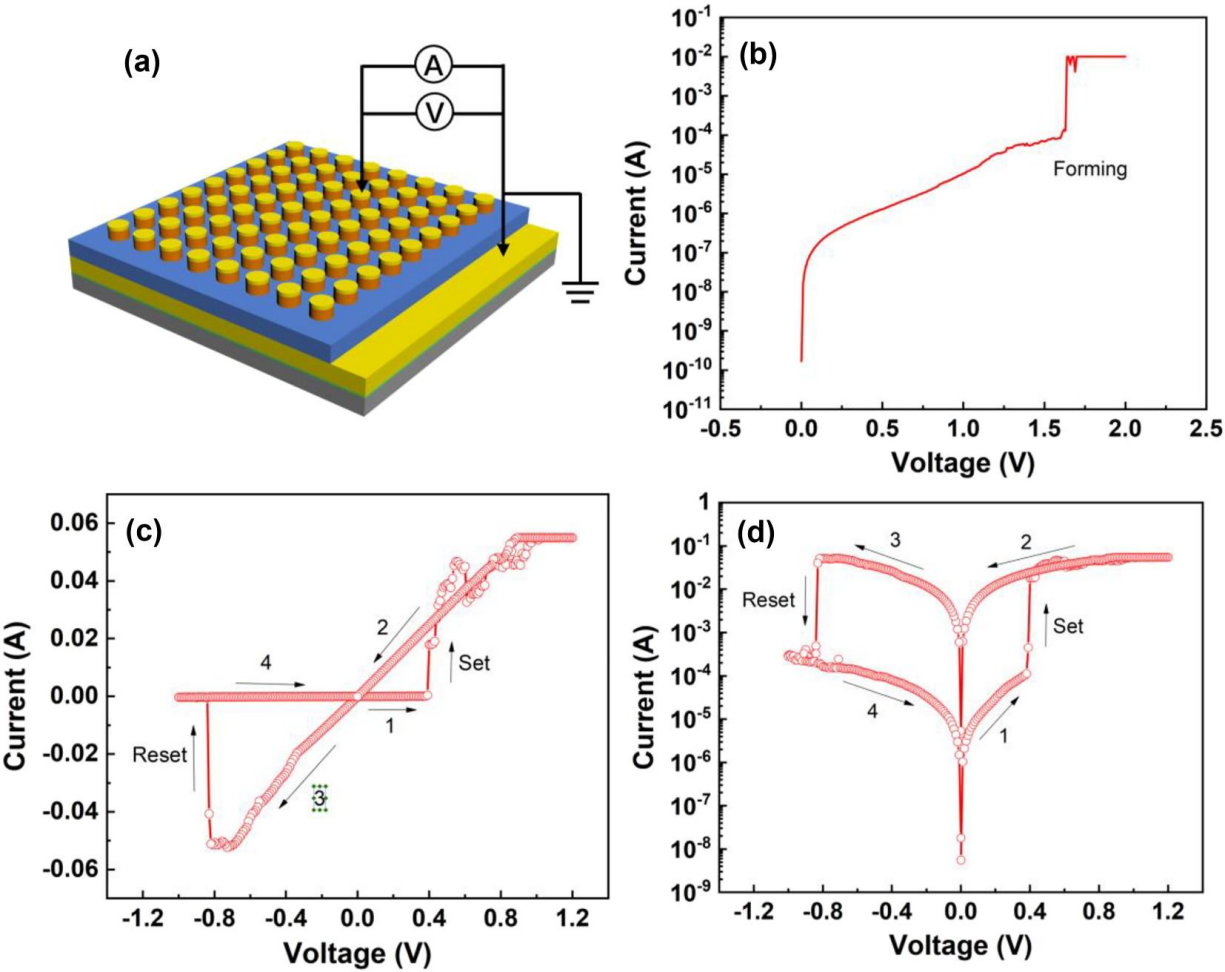
(a) Schematic configuration of the NbS_2_ CBRAM devices studied in this work; (b) the I‐V curve of the device during the forming process. The measured I‐V curves in linear (c) and logarithmic coordinates (d). CBRAM, conductive‐bridge random access memory.

A schematic configuration of the two‐terminal devices studied in this work is depicted in Figure 3a. The Cu/NbS_2_/Au device fabricated in the experiment has a typical two‐terminal structure. During the measurement, the bottom electrode is grounded, electric excitation is applied between the top electrode and the bottom electrode through a Keithley 4200 semiconductor parameter analyzer, and the change in the resistance value of the device is monitored. For the sake of simplicity, we stipulate that the positive direction of the voltage or current is the direction of the Cu top electrode relative to the Au bottom electrode.

The resistive current‐voltage (I‐V) curve of the Cu/NbS_2_/Au CBRAM device was measured by the voltage scanning mode in the experiment, and the scanning step is generally 0.01 V. The initial device exhibits a high resistance state (HRS). To switch the device into the low resistance state (LRS), a forming process with a forming voltage of up to 1.6 V is required, as shown in Figure 3b. The measured I‐V curves are displayed in linear and logarithmic coordinates in Figure 3c,d, respectively, and the numbers show the sequence of voltage scans during the measurement process, that is, 0 V—1.2 V—0 V—−1 V—0 V. The Cu/NbS_2_/Au device in the initial state was in a HRS with resistance as high as 10^4^ Ω. When a forward voltage is applied to the device and gradually increases, the resistance of the device remains unchanged at first, and then suddenly changes at about 0.5 V, and the resistance reduces from 10^4^ Ω to about 10 Ω, which is called “set” process. During the voltage sweep from 1.2 V back to 0, the device resistance remains at an LRS of 10 Ω, which proves that the LRS of the device is non‐volatile. In order to change the device from a LRS to a HRS, a negative voltage of about −0.8 V needs to be applied to the device, and the resistance of the device elevates form 10 Ω–10^4^ Ω, which is called “reset” process. As shown in Figure 3c, during the voltage sweep from −1 V back to 0, the device remains in the HRS, showing that the HRS is also non‐volatile. The absolute values of set voltage (V_set_) and reset voltage (V_reset_) are both lower than 1 V, which are lower than most reported RRAM devices, meaning that NbS_2_ CBRAM can operate at low voltage. Low‐voltage operation reduces power consumption and allows the device to be used in more advanced CMOS processes. Since the Set process of the device is completed under positive voltage conditions, and the Reset process is completed under negative voltage conditions, the experimentally prepared Cu/NbS_2_/Au RRAM exhibits typical bipolar RRAM characteristics. According to literature reports, the resistance transition effect controlled by Joule heat does not show voltage polarity, so it can be guessed that the Resistive switching of Cu/NbS_2_/Au CBRAM is probably controlled by electrochemical redox reaction.

**FIGURE 4:**
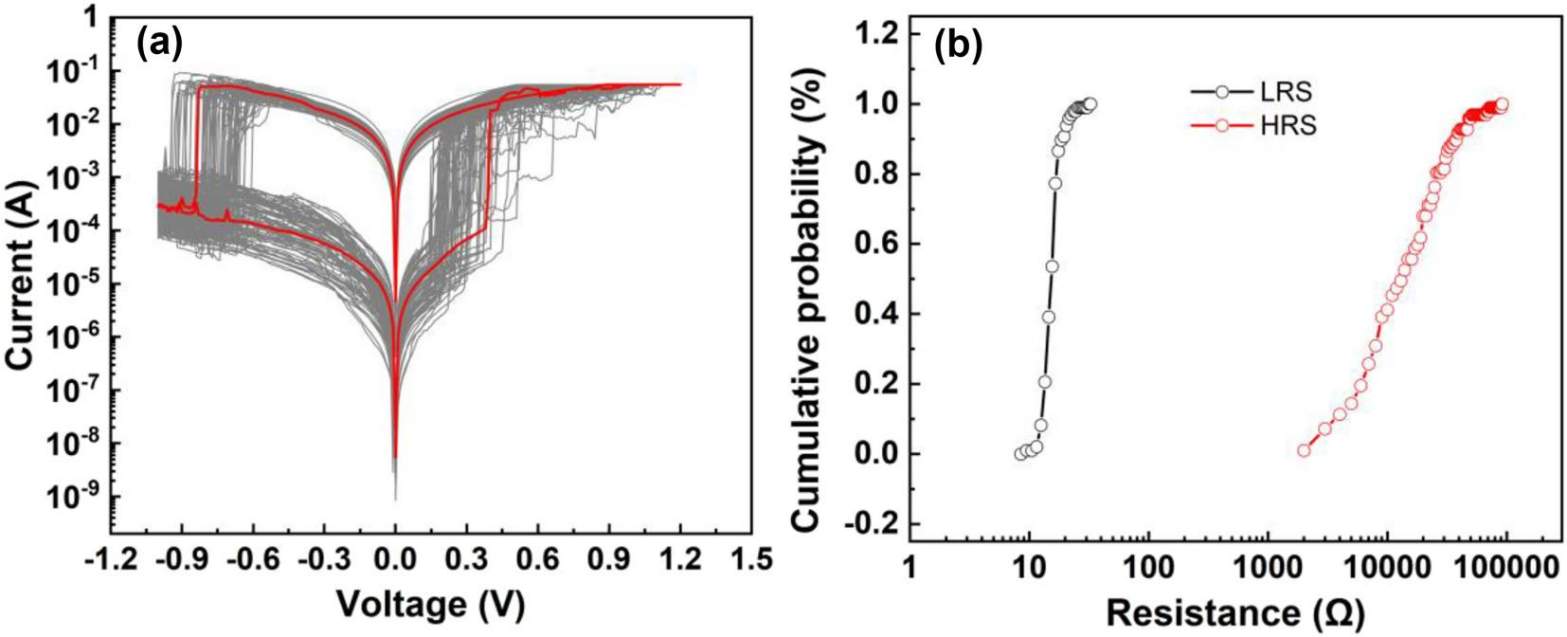
(a) Resistive switching I–V curves of the NbS_2_ CBRAM device under 100 repeated cycles; (b) the statistical distribution of the HRS and LRS resistances of the CBRAM device during the repeated resistive switching process. CBRAM, conductive‐bridge random access memory; HRS, high resistance state; LRS, low resistance state.

In order to verify the NbS_2_ CBRAM device can realize the RS repeatedly, we tested the RS characteristics of the device in the scanning voltage mode for multiple cycles. The voltage change in a cycle is 0 V—1.2 V—0 V—−1 V—0 V. Figure 4a shows the I–V curves of the device under 100 repeated cycles. The results show that the NbS_2_ devices can successfully transition between high‐resistance and low‐resistance states, exhibiting good cycling stability. The distribution ranges of V_set_ and V_reset_ of NbS_2_ CBRAM are from −0.95 to 0.65 V and 0.2–0.5 V. Although the set voltage and reset voltage of the device fluctuate to a certain extent, the operating voltage during all RS cycles is less than 1 V. As a result, a CBRAM device with low voltages has been demonstrated. Figure 4b shows the statistical distribution of the HRS and LRS resistances of the CBRAM device during the repeated RS process. The distribution ranges of HRS resistance and LRS resistance of NbS_2_ CBRAM are from 2500 to 90,000 Ω and 9–25 Ω. The resistance distribution of the high and LRS resistance is relatively concentrated, the fluctuation is not large, the on‐off ratio has been kept above 100, and the device has a large enough data reading window.

**FIGURE 5:**
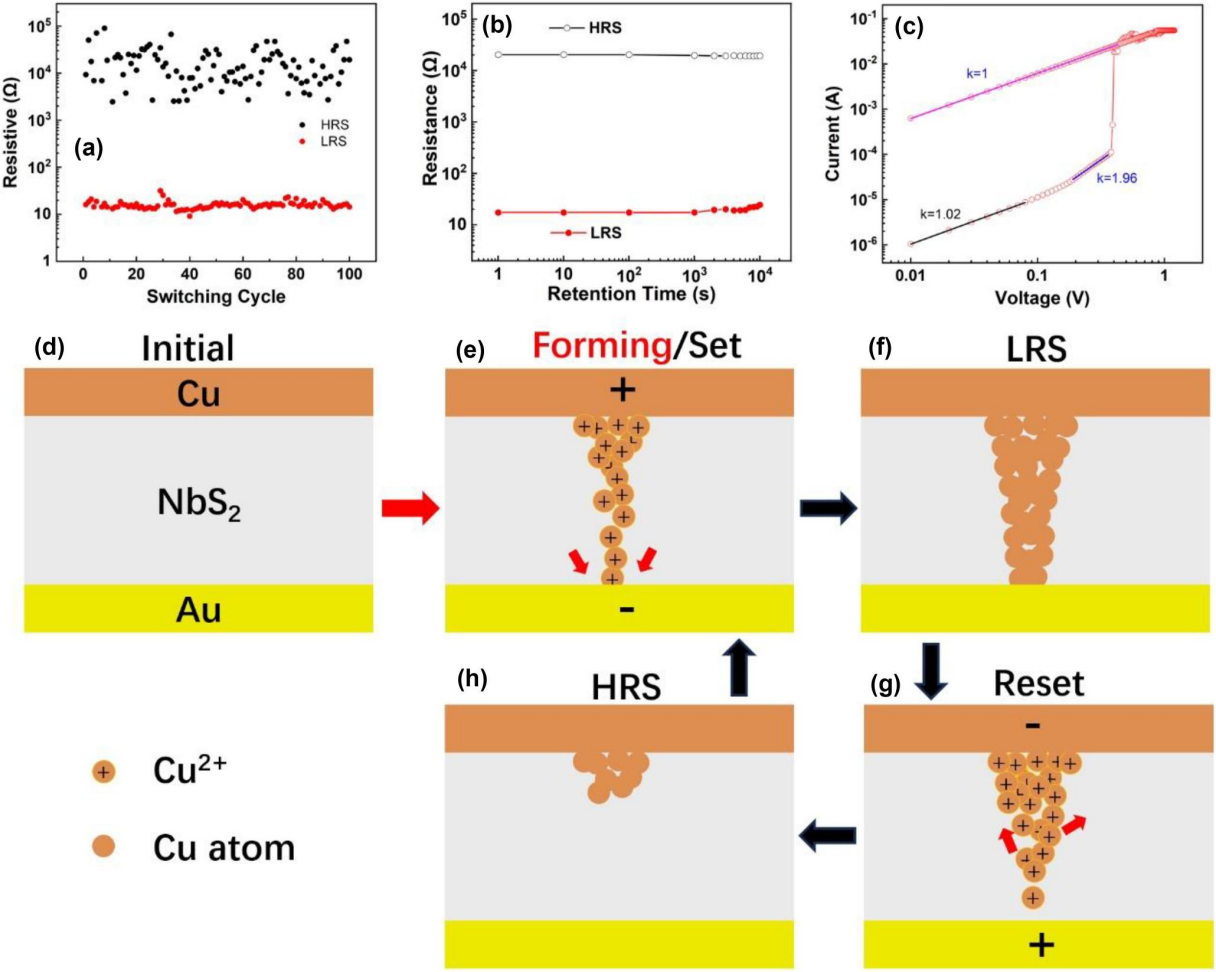
(a) The endurance characteristic of the NbS_2_ CBRAM device; (b) the retention characteristics of the NbS_2_ CBRAM device at HRS and LRS; (c) I‐V characteristics with *x* and *y* axes in logarithmic coordinates during the set process, with linear fitting curves to the curves for the HRS and LRS; Schematic of resistive switching mechanism: (d) initial device, (e) forming or set process, (f) device in LRS, (g) reset process, (h) device in HRS. CBRAM, conductive‐bridge random access memory; HRS, high resistance state; LRS, low resistance state.

Figure 5a shows the endurance characteristics of the device. The NbS_2_ CBRAM devices demonstrated stable endurance of up to 100 cycles. The resistance distributions are very narrow suggesting good uniformity of HRS and LRS. Figure 5b displays the retention characteristics of the NbS_2_ CBRAM device at HRS and LRS. The resistance value of the device was read by a V_read_ of 0.01 V every 1 s. The device exhibited uniform resistance for HRS and LRS over 10^4^ s, showing good retention performance.

In order to study the RS mechanism of NbS_2_ CBRAM, we converted the resistance transition I‐V curve in Figure 3c into log‐logarithmic coordinates (logI‐logV curve), as shown in Figure 5c. Piecewise linear fitting was performed on the logI‐logV curve and the corresponding slope was calculated to further analyze the dependence of current and voltage. It can be seen from the logarithmic curve that the HRS and LRS of the device show different current‐voltage dependencies, indicating that the conduction mechanism of the low‐resistance state of the device is inconsistent with that of the high‐resistance state. In general, the conduction mechanism of CBRAMs based on metal CFs in the high‐resistance state conforms to space‐charge‐limited conduction (SCLC).[^32^, ^33^] Specifically, in the low‐voltage region, the voltage and current satisfy Ohm's law, and the slope of the straight line fitted to the logI‐logV curve is 1; as the voltage increases, the relationship between voltage and current satisfies Child's law, and the slope of the fitted line will change to ∼2 when the voltage increases, which is called the trap‐unfilled SCLC region; when the voltage further increases, the current increases sharply with the voltage, and the slope of the curve increases to ∼5, which is called the trap‐filled SCLC region. For the HRS of a‐NbS_2_ CBRAM, the slope of the fitted straight line of logI‐logV increases from 1.03 to 1.96 during the set process, indicating that the HRS of our device fits well with the SCLC mechanism. For the low‐resistance state of a‐NbS_2_ CBRAM, the slope of the fitted curve is exactly 1, showing a complete ohmic conduction mechanism, which also indicates the formation of Cu CFs in the NbS_2_ resistive switch layer. Not necessarily devices contain all three regions. In our NbS_2_ CBRAM, during set progress, the V_set_ is so tiny that the device switches from HRS to LRS when the I‐V curve is still in the Child's law region.

Figure 5d–h illustrates a schematic diagram of the switching mechanism. In Cu/NbS_2_/Au CBRAM devices, the migration of Cu ions under an electric field leading to the formation and rupture of Cu CFs is considered to be the main mechanism of device resistance change, and the specific process is as follows: (1) At first, the initial device exhibits HRS (Figure 5d), a forming voltage will switch the device to LRS; (2) The top electrode is positively biased, and the bottom electrode is grounded, the copper electrode undergoes electrochemical anodic dissolution according to the reaction Cu → Cu^2+^ + 2e^−^; (3) Due to the amorphous nature of NbS_2_, Cu ions migrate into the a‐NbS_2_ resistive layer at a lower bias (Figure 5e); (4) Cu ions reach The bottom electrode, according to Cu^2+^ + 2e^−^ → Cu reaction, copper ions are reduced and electrocrystallized. The electrocrystallization process leads to the formation of metal CFs, the memory cell switches to LRS (Figure 5f); (5) The upper electrode is negatively biased, the lower electrode is grounded, and the Cu CF is formed due to the electrochemical dissolution of Cu. break, the device switches to HRS, and a Reset operation occurs (Figure 5g,h).

## 4 CONCLUSION

In summary, NbS_2_ films were prepared by magnetron sputtering at room temperature. The NbS_2_ films have a smooth surface, uniform thickness, and even distribution of elements. NbS_2_ films exhibit an amorphous structure and do not crystalize even annealed at 500°C, showing high thermal stability. The amorphous NbS_2_ CBRAM devices present stable bipolar non‐volatile RS characteristics with an operation voltage below 1 V and HRS/LRS resistance ratio of 100. Repetitive RS behavior is demonstrated in amorphous NbS_2_ CBRAM devices. The electrochemical redox reaction of Cu as well as the formation and fracture of Cu metal CF is considered to be the RS mechanism. In conclusion, NbS_2_ is a promising material for low‐operation voltage CBRAM devices.

## CONFLICT OF INTEREST STATEMENT

The authors declare no conflicts of interest.

## ETHICS STATEMENT

This research does not involve any human participants or animals.

## Data Availability

The data that support the findings of this study are available from the corresponding author upon reasonable request.
